# Identity preserved plant molecular farming offers value-added opportunity for farmers

**DOI:** 10.3389/fpls.2024.1434778

**Published:** 2024-06-19

**Authors:** Kyle Kunkler, Scott Gerlt

**Affiliations:** ^1^ Department of Government Affairs, American Soybean Association, Washington, DC, United States; ^2^ Department of Economics, American Soybean Association, St. Louis, MO, United States

**Keywords:** molecular farming, soybeans, farming, biotechnology, stewardship, recombinant proteins, high oleic

## Abstract

Bulk commodity row crop production in the United States is frequently subject to narrow profit margins, often complicated by weather, supply chains, trade, and other factors. Farmers seeking to increase profits and hedge against market volatility often seek to diversify their operations, including producing more lucrative or productive crop varieties. Recombinant plants producing animal or other non-native proteins (commonly referred to as plant molecular farming) present a value-added opportunity for row crop farmers. However, these crops must be produced under robust identity preserved systems to prevent comingling with bulk commodities to maintain the value for farmers, mitigate against market disruptions, and minimize any potential food, feed, or environmental risks.

## 1 Introduction

U.S. farmers are generally price takers in agricultural commodity markets. In these competitive markets, farmers operate on narrow margins and normally must accept the prevailing market price for their crops. This pressure is especially intense for row crop farmers, like soybean growers, who sell into bulk commodity markets and have limited value-added farming opportunities or options for direct marketing to boost farming revenues. Soybean farmers face these market conditions regardless of the variability of production costs or other factors that can affect the market or their individual farming operations. In many instances, U.S. farm revenues do not exceed the cost of production.

Farmers are often willing to consider innovative solutions to support the continued viability of their operations. If they have alternative options to supplement or diversify their farming operation to augment income, many will pursue those opportunities. Recombinant crop varieties producing novel proteins, often referred to as plant molecular farming (PMF), present an exciting new possibility for farmers to produce a value-added product relative to bulk commodity crops, likely boosting income. Despite these opportunities, growing new PMF varieties may also present several risks and challenges. So long as these challenges are carefully managed to prevent market or safety concerns and the financial incentive for production outweighs costs and burdens, farmers will welcome this new opportunity.

## 2 Evidence from high oleic soybean varieties

In recent years, U.S. soybean farmers have produced several specialty soybean varieties that can serve as case studies for the potential value-added opportunity that PMF could bring to U.S. farmers and markets. One of the most insightful examples is soybeans developed to produce high oleic soybean oil (HOSBO). Soybeans are an oilseed crop, the oil from which is commonly extracted and used for cooking and frying, among other uses. Conventional soybean oil contains higher levels of linoleic acid, which has a greater tendency to oxidize and reduce the shelf-life for cooking oils. HOSBO contains reduced levels of linoleic acids and significantly higher levels of oleic acids, which are not only more shelf-stable but also have been found to reduce the risks of coronary heart disease relative to consuming other edible oils (Lichtenstein et al., 2006; Fehr, 2007; Baer et al., 2021). These characteristics carry value for consumers and food processors, who are likely to pay a premium for farmers to produce HOSBO for food uses. To address these market opportunities, several recombinant varieties of soybeans have been developed to produce HOSBO, though several high oleic varieties also have been produced using genome editing or other conventional breeding techniques.

Following the limited launch of high oleic in 2010 after U.S. regulatory approvals were granted, adoption of high oleic soybean acres grew slowly at first, limited by several factors. Recombinant high oleic varieties did not initially have the export approval of several major international markets for U.S. soybeans. Obtaining export approval from major international markets for novel recombinant varieties can be an important step for preventing what could be significant disruptions to the broader commodity market, as discussed further below. Without these export market approvals, supplies must be stewarded carefully in closed-loop, identity preserved (IP) systems to prevent comingling with bulk commodity varieties. The labor-intensive steps needed to maintain these IP systems (segregating crops, equipment cleaning, monitoring for volunteer plants, etc.) can serve as a barrier for adoption by farmers, who are likely unwilling to grow these varieties unless compensated for their efforts.

China, the largest importer of U.S. soybeans, did not grant export approval for soybeans containing the high oleic trait until 2011 (Anderson-Sprecher and Jie, 2014), and the European Union (EU) did not grant its first export approval until 2015 (European Commission, 2015). EU approval of the high oleic trait stacked with a glyphosate-tolerance trait for weed management did not come until December 2017 (European Commission, 2017). Addressing herbicide tolerance was another vital step for enabling U.S. adoption, as many farmers would be reluctant to produce high oleic varieties without meaningful herbicidal weed control options. Weeds can significantly reduce soybean crop potential, causing more than 50 percent yield loss if left uncontrolled (Soltani et al., 2018). Without sufficient weed management options, any premium gained from producing specialty soybean varieties could be offset by yield loss from weeds if that yield loss is not already accounted for in premiums. While many weed populations have developed resistance to glyphosate, the availability of the glyphosate-tolerant trait gives farmers some means to address weeds following crop emergence.

Risk management and agronomic viability are important considerations for farmers when determining whether to produce specialty varieties (Bard et al., 2003). Strengthening these characteristics and attaining export market approvals helped increase farmer interest in growing high oleic varieties, yet other factors remained that needed to be addressed to ensure market sustainability. These considerations included making certain there was sufficient crop storage and processing capacity to prevent comingling with bulk commodity soybeans (United Soybean Board, 2023).

For high oleic varieties, these robust IP segregation requirements waned somewhat after major export market approvals were attained. The risks of potential disruptions from export markets detecting an unapproved biotechnology variety were alleviated, and in many cases, HOSBO could be blended with conventional soybean oil to reach targeted oleic acid levels. Most of the continued need for crop segregation comes from preserving crop value for farmers and ensuring there is sufficient high oleic soybean supplies to meet market demand. While this reduced IP burden has been beneficial for high oleic soybean adoption, we expect it will not be possible for all specialty PMF soybean varieties, as discussed below. However, to maintain more rigorous IP systems would require greater costs and time requirements for farmers maintaining these protocols, which may translate to greater premiums to incentivize farmers to produce those varieties.

While the journey for widespread adoption of high oleic soybeans has been lengthy, progress has been made. High oleic soybean varieties are expected to surpass more than one million acres in the U.S. for the first time in 2024 (United Soybean Board, 2023). In 2023, grain elevators paid premiums between $0.95-$2.05 per bushel for high oleic soybeans. For a farmer producing 50 bushels of soybeans per acre with yields comparable to conventional soybeans, this could represent a $102.50 production premium per acre. In this scenario, producing high oleic varieties on 500 acres would represent a $51,250 premium relative to growing conventional soybeans.

## 3 Plant molecular farming versus precision fermentation

Before considering the feasibility of growing soybeans for PMF, we must first consider the cost of production of PMF relative to tank precision fermentation processes using mammalian or microbial cells, another technology for producing recombinant proteins. There is ample evidence to suggest PMF can be a cost-effective alternative. One analysis found generating recombinant monoclonal antibodies (mAB) in corn plants could result in a two-thirds reduction in the production cost per unit compared to a mammalian cell facility (Kaufman and Kalaitzandonakes, 2011). Another found the cost of producing a lifetime supply of mABs in tobacco for the treatment of non-Hodgkin lymphoma could be as low as $15,000—a ten-fold reduction compared to other conventional systems (Chung et al., 2022).

It should be noted that costs for PMF can vary highly and not all applications will be economically feasible. Two analyses producing recombinant proteins in tobacco identified an order of magnitude difference between the cost of the finished product (Schillberg and Finnern, 2021). The factors affecting the potential cost are numerous, including research and development; regulatory compliance; scale of production; capital investment in refinement and processing facilities; protein expression levels in the host plant; crop inputs, production, and harvesting; uses for remaining crop biomass; labor and transportation; among others (Kaufman and Kalaitzandonakes, 2011; Buyel, 2019). These variables all must be considered in the feasibility of producing a recombinant protein using PMF or some other process.

While the concept of PMF for recombinant protein production has been considered for decades, large-scale operations have been few to date, in great part because most of the applications contemplated are biomedical in nature. As one biopharmaceutical product manager noted, “The biopharma world doesn’t traditionally focus on cost of goods because they are relatively small compared to their sale prices. And, biopharma sales prices are high to cover all the R&D, regulatory, and other costs specifically related to biopharma production” (Dutton, 2023). When the costs of production are minimal relative to the price of the finished product, there is little need or incentive to transition from a predictable, effective production system such as precision fermentation, especially when there are many novel variables to consider with PMF (environmental factors, contracting with farmers, etc.).

Food and feed applications, however, are different. The finished product of recombinant proteins intended for human or animal consumption typically carries a much lower market price relative to pharmaceutical products. This is especially relevant if the recombinant food or feed product is attempting to compete in a market already saturated by a relatively low-cost conventional agricultural product. We observe that recent growth in interest in PMF applications has largely come from these potential food and feed applications where the cost of production greatly affects the price of the finished product. Soybeans have much to offer many of these possible applications.

## 4 Possibilities for PMF specialty soybean varieties

Soybeans have exceptional potential to serve as a platform for PMF of recombinant proteins and have numerous benefits relative to other crops for this purpose. An initial advantage of soybeans is that they are high in protein content and are one of the few commercially available crops that naturally contain all nine essential amino acids necessary for human nutrition (Michelfelder, 2009). On average, 100 grams of defatted soybean meal contains 49.2 grams of protein (Agricultural Research Service, 2019). For comparison, 100 grams of corn meal on average contains 6.2 grams of protein (Agricultural Research Service, 2020), while 100 grams of chicken breast contains 22.5 grams of protein (Agricultural Research Service, 2023). This high protein content makes soybeans an excellent animal feed, which is one of the primary purposes of soybean meal. As discussed above, soybeans are also an oilseed and contain vital dietary fats, the extraction of which is a common source of cooking oil. Globally, soybean oil is one of the largest sources of edible oils for cooking and food processing (Food and Agriculture Organization, 2023).

The academic, crop development, and farming communities also have extensive experience using genetic improvement techniques in soybeans. In the U.S., genetically engineered soybeans were first introduced in 1996 and now cover at least 95 percent of soybean acres, largely containing herbicide-tolerant crop traits (Economic Research Service, 2023). This familiarity within the research, development, and production communities facilitates an environment for the eased introduction of other recombinant crop varieties relative to other crops without a history of use of genetic engineering.

Given that soybeans already serve as a significant food and feed staple globally and have a long history of producing recombinant varieties, the use of soybeans for PMF is intuitive, especially where the finished product is intended for food or feed purposes. The production of recombinant proteins in soybeans can enhance a soy-based food or feed product to better fit its intended market use. For example, limited concentrations of total sulfur amino acids (TSAA) in the diets of broiler chickens can significantly limit feed conversion ratio and body weight growth of poultry (Farkhoy et al., 2012). However, a recombinant soybean variety (M703) developed to produce total higher levels of TSAA was identified as having potential to improve dietary outcomes for broiler chickens by providing greater access to these vital amino acids (Edwards et al., 2000).

Another example is soybean plants that have been modified to express recombinant bovine casein proteins (Philip et al., 2001). There is significant food application interest in this innovation for producing more realistic, soy-based alternatives to traditional dairy cheeses. Since soybeans do not ordinarily produce casein, a protein essential for achieving soft, cohesive textures and other important functional attributes in dairy cheeses (Kim (Lee) et al., 1992), manufacturers of soy-based alternatives currently must add refined casein from dairy cows during the production process. However, the use of dairy casein can prevent manufacturers from making vegan or other marketing claims and thus may limit the number of consumers for those products. If the soybeans were to produce recombinant casein proteins directly, it would enable manufacturers to produce more realistic, soy-based alternatives and allow for market claims they otherwise could not make.

It is worth noting that in both instances above, the initial product is soy-based and then its market potential is enhanced through the introduction or greater expression of recombinant proteins. Additional processing or extraction of recombinant proteins is minimal or may not be required at all. This could be an important consideration for food and feed applications of PMF to minimize processing costs. In one of the previously referenced examples of mAB production in tobacco, downstream processing and extraction of the refined protein accounted for 84 percent of the total manufacturing costs (Schillberg and Finnern, 2021). Identifying ways to minimize downstream production or refinement costs, such as retaining recombinant proteins in a finished soy-based product, may be a vital approach for allowing applications of PMF to flourish in food or feed markets where profit margins are narrow and costs could be constrained by competition with conventional agricultural goods.

## 5 Regulatory, cultural, market, and other considerations

There are exciting prospects for PMF in soybeans; however, depending upon the type of innovation, there are also numerous potential economic, environmental, and human health factors that must be considered. One of the most pressing concerns is that of possible allergenicity. When introducing novel recombinant proteins into a host organism, there is a risk that those proteins may pose a food or feed allergenicity risk. These risks are heightened when the donor organism of recombinant proteins is a known allergen. In 2023, the U.S. Food and Drug Administration (FDA) issued a public letter raising concerns with the development of novel recombinant allergens for PMF purposes, including steps that the stakeholder community should take to assess and minimize these risks (Food and Drug Administration, 2023).

Potential market disruption risks have long been another concern of commercializing recombinant biotechnology traits and have materialized in several instances, one of which stemmed from the commercialization of a recombinant insect control trait in corn by Aventis Crop Science. The variety, known by the trade name StarLink, expressed recombinant Cry9c proteins from *Bacillus thuringiensis* (Bt). The U.S. Environmental Protection Agency (EPA) approved Cry9c expression only for use in feed crops, as the agency had lingering questions about potential protein allergenicity risks in humans (Lin et al., 2002). Despite this restriction, Cry9c proteins were detected in late 2000 in taco shells in the U.S. and then in other products containing corn intended for human consumption. Detections of Cry9c in U.S. corn shipments to Japan (which had a zero-tolerance policy for StarLink presence since the trait had not received export approval from Japan) and Korea led to corn exports being suspended temporarily by those countries. One estimate places the economic impact of the Starlink disruption to U.S. farmers at between $26 million and $288 million in lost revenue (Schmitz et al., 2005).

A concern not yet experienced in recombinant protein development is that of potential market disruptions due to cultural concerns. Looking at global implications, soybeans are not subject to dietary restrictions by any major cultural or religious group and thus are not a major consideration for market certification. However, if recombinant proteins were introduced into host plants from donor organisms to which major cultural or religious groups have food restrictions, crops may be required to attain food certifications for market access (kosher, halal, etc.) or risk disruption.

These scenarios pose various potential PMF risks that could result in significant harm to consumers, agricultural markets, or individual farming operations. While the risks are unique to the individual product and its intended market use, many of the potential safeguards are similar and can be customized to fit the risk profile of the innovation. The first and most important protection is engaging with regulators to acquire all necessary regulatory approvals. These processes will help identify all potential risks and how to best mitigate them.

A robust IP system for PMF crop varieties will likely be essential for stewarding many innovations. If implemented properly, these closed loop systems could prevent comingling PMF varieties with bulk commodities, greatly minimizing the risks discussed above. IP systems also will be vital for farmers in protecting production premiums of PMF varieties. In the instance of food safety risks from PMF, IP processes would need to adopt a zero-tolerance approach and add duplicative layers of protection to prevent system failure. While these IP systems will add to the cost of PMF production, they will be essential for protecting farmers, consumers, and the continued viability of markets.

Another protection PMF developers should consider is attaining export market approvals for all major markets to which a bulk commodity crop is exported, even if that country is not the intended market for the PMF variety or refined recombinant proteins. In the event of a breach of an IP system, this protection could prevent market disruptions and potential legal risk to PMF developers.

## 6 Discussion

U.S. soybean farmers are generally supportive of innovative new products and markets, especially if these opportunities can add value to the goods they produce and can boost farm incomes. U.S. growers have faced farm income pressures for decades and always seek ways to enhance the viability of their operations. PMF has immense potential to offer these opportunities—not just for the farmers who will help grow them but also consumers and end users who stand to benefit from new solutions that address market needs.

Soybeans have numerous characteristics that make them ideal for developing many of these new products. As one of the largest acreage crops in the U.S., soybeans already have significant domestic and global market share for edible oil and proteins; this is in large part due to their favorable nutritional profile and a reliable infrastructure system for getting them to market. Using soybeans as bioreactors to produce recombinant proteins, either directly for protein extraction or for enhancing the composition of the soybeans to better address specific market needs, is a practical consideration for this versatile crop.

Additionally, the U.S. soybean research, development, and producer community has a long history with using genetic engineering for plant enhancement, having been one of the first crops to adopt these tools. This experience has provided valuable lessons about how to best steward these products to preserve their value and prevent potential risks from their uses.

As eager as farmers likely will be to partner with PMF developers to produce these exciting new innovations, it is however essential that any risks individual products could pose are carefully managed. For the foreseeable future, U.S. soybeans will remain a bulk commodity market, relying on nearly $28 billion in exports annually (Foreign Agricultural Service, 2024). The detection of unapproved biotechnology events by trade partners could not only create immediate market turmoil, but also it would likely erode U.S. global market share long-term if overseas customers were forced to go elsewhere for reliable supplies. Further, potential human health, animal, or environmental risks posed by specific innovations could undermine public confidence in U.S. agricultural production, our food supply, and genetic innovation. Acquiring necessary regulatory approvals and establishing meaningful IP systems are the most appropriate, effective ways to manage these risks.

Assuming these challenges can be managed reliably, U.S. soybean farmers are ready for the prospects that PMF can bring to agriculture and consumers. The applications for this technology are vast, which presents great opportunities for innovators, farmers, and the markets they serve.

## Data availability statement

The original contributions presented in the study are included in the article/supplementary material. Further inquiries can be directed to the corresponding author.

## Author contributions

KK: Writing – original draft, Writing – review & editing. SG: Writing – review & editing.
